# Steering Witness and Steering Criterion of Gaussian States

**DOI:** 10.3390/e24010062

**Published:** 2021-12-29

**Authors:** Ruifen Ma, Taotao Yan, Dantong Wu, Xiaofei Qi

**Affiliations:** 1 Department of Mathematics, Taiyuan University of Science and Technology, Taiyuan 030024, China; ruifenma@tyust.edu.cn (R.M.); S20201801034@stu.tyust.edu.cn (T.Y.); S20201801029@stu.tyust.edu.cn (D.W.); 2 School of Mathematical Science, Shanxi University, Taiyuan 030006, China

**Keywords:** quantum EPR steering, steering witness, steering criterion, covariance matrix, gaussian states

## Abstract

Quantum steering is an important quantum resource, which is intermediate between entanglement and Bell nonlocality. In this paper, we study steering witnesses for Gaussian states in continuous-variable systems. We give a definition of steering witnesses by covariance matrices of Gaussian states, and then obtain a steering criterion by steering witnesses to detect steerability of any (m+n)-mode Gaussian states. In addition, the conditions for two steering witnesses to be comparable and the optimality of steering witnesses are also discussed.

## 1. Introduction

Entanglement plays an important role in quantum information theory, which has been widely used in quantum information processing [1,2,3]. The detection of entanglement has attracted much attention in recent years (see [4,5,6,7,8,9,10,11,12,13,14,15]). Among these criteria, the entanglement witness (EW) criterion provides a sufficient and necessary condition for the separability of a bipartite quantum state ([5]). A self-adjoint operator *W* acting on a separable complex Hilbert space H⊗K is an EW if *W* is not positive and Tr(Wσ)≥0 holds for all separable states σ. It was shown that a bipartite state is entangled if and only if there exists at least one EW detecting it. Obviously, there does not exist an EW that can detect all entangled states. So, the concept of the optimal EW is proposed in [8] and some methods are given to check the optimality of EWs, for example, see [8,11,12,15].

In 1935, Einstein, Podolsky and Rosen (EPR) first discovered the anomalous phenomenon of quantum states in multipartite quantum systems, which is contrary to the classical mechanics ([16]). In order to capture the essence of the EPR paradox, the notion of EPR steering was first introduced by Schr$\ddot{o}$dinger in [17]. EPR steering is a quantum correlation between entanglement and Bell nonlocality. Different from entanglement and nonlocality, this correlation is inherently asymmetry with respect to the observers.

In recent years, EPR steering has attracted many authors’ attention. It has been shown that EPR steering plays a fundamental role in various quantum protocols, secure communication, and other fields ([18,19,20]). Various EPR steering criteria have been derived. For example, Cavalcanti and James in [21] obtained the experimental criterion of EPR steering from entropy uncertainty relations. Ji et al. in [22] obtained steerability criteria by using covariance matrices of local observables, which are applicable for both finite- and infinite-dimensional quantum systems. Wittmann et al. in [23] gave EPR steering inequalities with three Pauli measurements; and then, as a generalization of the Pauli matrices, Marciniak et al. in [24] found EPR steering inequalities with mutually unbiased bases. For continuous-variable systems, the authors in [25] performed a systematic investigation of EPR steering for bipartite Gaussian states by pseudospin measurements. Kogias and Adesso [26] gave a measure of EPR steering for two-mode continuous variable states.

Inspired by EW, in this paper, we will try to consider quantum EPR steering witness for Gaussian states in continuous-variable systems. This paper is organized as follows. In Section 2, we recall the concepts of Gaussian states and quantum EPR steering, and some known EPR steering criteria for Gaussian states. Section 3 is devoted to giving a definition of steering witness for Gaussian states in terms of covariance matrices and then discussing properties of steering witness. Based on steering witnesses, a steering criterion for bipartite (m+n)-mode Gaussian states are obtained. The conditions for two steering witnesses to be comparable are given and the optimality of steering witnesses are also obtained. Section 4 is a brief conclusion.

## 2. Definition and Criterion of Gaussian Quantum EPR Steering

In this section, we briefly introduce the notion of Gaussian quantum steering.

*Gaussian states.* Recall that a quantum system is associated with a separable complex Hilbert space *H*. A quantum state on *H* is a positive operator with trace 1. For arbitrary state ρ in an *n*-mode continuous-variable system with state space *H*, its characteristic function $\chi_{\rho }$ is defined as

$$\chi_{\rho }\left(z\right)=\mathrm{tr}\left(\rho W\left(z\right)\right),$$

where $z={\left(x_{1},y_{1},\cdots ,x_{n},y_{n}\right)}^{T}\in R^{2n}$, $W\left(z\right)=\exp\left(iR^{T}z\right)$ is the Weyl displacement operator, $R=\left(R_{1},R_{2},\cdots ,R_{2n}\right)=\left({\hat{Q}}_{1},{\hat{P}}_{1},\cdots ,{\hat{Q}}_{n},{\hat{P}}_{n}\right)$. As usual, $\hat{Q_{k}}=\left({\hat{a}}_{k}+{{\hat{a}}_{k}}^{†}\right)/\sqrt{2}$ and $\hat{P_{k}}=-i\left({\hat{a}}_{k}-{{\hat{a}}_{k}}^{†}\right)/\sqrt{2}$ (k=1,2,⋯,n) stand for, respectively, the position and momentum operators, where ${\hat{a}}_{k}^{†}$ and ${\hat{a}}_{k}$ are the creation and annihilation operators in the *k*th mode, satisfying the Canonical Commutation Relation (CCR)

$$\left[{\hat{a}}_{k},{\hat{a}}_{l}^{†}\right]=\delta_{kl}I\;\mathrm{and}\;\left[{\hat{a}}_{k}^{†},{\hat{a}}_{l}^{†}\right]=\left[{\hat{a}}_{k},{\hat{a}}_{l}\right]=0,\;\;k,l=1,2,\cdots ,n.$$

Particularly, ρ is called a Gaussian state if $\chi_{\rho }\left(z\right)$ is of the form

$$\begin{array}{c} \chi_{\rho }\left(z\right)=\exp\left[-\frac{1}{4}z^{T}\Gamma z+id^{T}z\right], \end{array}$$

where

$$d={\left(\langle {\hat{R}}_{1}\rangle ,\langle {\hat{R}}_{2}\rangle ,\ldots ,\langle {\hat{R}}_{2n}\rangle \right)}^{T}={\left(\mathrm{tr}\left(\rho R_{1}\right),\mathrm{tr}\left(\rho R_{2}\right),\ldots ,\mathrm{tr}\left(\rho R_{2n}\right)\right)}^{T}\in R^{2n}$$

is called the mean or the displacement vector of ρ and $\Gamma =\left(\gamma_{kl}\right)\in M_{2n}\left(R\right)$ is the covariance matrix (CM) of ρ defined by $\gamma_{kl}=\mathrm{tr}\left[\rho \left(\Delta {\hat{R}}_{k}\Delta {\hat{R}}_{l}+\Delta {\hat{R}}_{l}\Delta {\hat{R}}_{k}\right)\right]$ with $\Delta {\hat{R}}_{k}={\hat{R}}_{k}-\langle {\hat{R}}_{k}\rangle$ ([27]). Here $M_{d}\left(R\right)$ stands for the algebra of all d×d matrices over the real field R. So, any Gaussian state ρ with CM Γ and displacement vector d will be represented as ρ(Γ,d). Note that Γ is real symmetric and satisfies the condition Γ+iJ≥0, where $J={⊕}_{k=1}^{n}J_{k}$ with $J_{k}=\left(\begin{array}{cc} 0 & 1\\ -1 & 0 \end{array}\right)$ for each *k*. Assume that $\rho_{AB}$ is any (m+n)-mode Gaussian state. Then its CM Γ can be written as

(1) $$\begin{array}{c} \Gamma =\left(\begin{array}{cc} A & C\\ C^{T} & B \end{array}\right),\;\;A\in M_{2m}\left(R\right),\;B\in M_{2n}\left(R\right),\;C\in M_{2m\times 2n}\left(R\right). \end{array}$$

*Qauntum steering.* Now let us recall the definition of steerability. A measurement assemblage $\mathrm{MA}={\left\{M_{a|x}\right\}}_{a,x}$ is a collection of positive operators $M_{a|x}\geq 0$ satisfying $\sum_{a}M_{a|x}=I$ for each *x*. Such a collection represents one positive-operator-valued measurement (POVM), describing a general quantum measurement, for each *x*. In a (bipartite) steering scenario, one party performs measurements on a shared state $\rho_{AB}$, which steers the quantum state of the other particle. If Alice performs a set of measurements ${\left\{M_{a|x}^{A}\right\}}_{a,x}$, then the collection of sub-normalized “steered states” of Bob are an assemblage ${\left\{\rho_{a|x}^{B}\right\}}_{a,x}$ with

$$\rho_{a|x}^{B}={\mathrm{Tr}}_{A}\left(\left(M_{a|x}^{A}⊗I_{B}\right)\rho_{AB}\right).$$

If every assemblage on Bob ${\left\{\sigma_{a|x}\right\}}_{a,x}$ can be explained by a local hidden state (LHS) model, of the form

$$\sigma_{a|x}=\sum_{\lambda }p_{\lambda }p\left(a|x,\lambda \right)\sigma_{\lambda },$$

where λ is a hidden variable, distributed according to $p_{\lambda }$, $\sigma_{\lambda }$ are “hidden states” of Bob, and p(a|x,λ) are local “response functions” of Alice, then we say that it has LHS form, or does not demonstrate steering ([28]). If there exist measurements such that $\sigma_{a|x}$ does not admit such an LHS decomposition, we say that the state $\rho_{AB}$ is steerable from A to B. If for all measurements we can never demonstrate steering with a given state, we say it is unsteerable from A to B. Symmetrically, we can define the steerability of $\rho_{AB}$ from B to A. Steering is a quantum correlation between entanglement and Bell nonlocality. However, unlike Bell nonlocality and nonseparability, which are symmetric between Alice and Bob, steering is inherently asymmetric.

*Gaussian Positive Operator-Valued Measurement.* An *m*-mode Gaussian Positive Operator-Valued Measurement (GPOVM) Π={Π(α)} is defined as

$$\Pi \left(\alpha \right)=\frac{1}{\pi^{m}}D\left(\alpha \right)\varpi D^{†}\left(\alpha \right),$$

where $D\left(\alpha \right)=\exp[\sum_{j=1}^{m}\left(\alpha_{j}{\hat{a}}_{j}^{†}-\alpha_{j}^{*}{\hat{a}}_{j}\right)]$ is the *m*-mode Weyl displacement operator, $\alpha \in C^{m}$, ϖ is a zero mean *m*-mode Gaussian state with CM Σ, which is called the seed state of the GPOVM Π. So, we can denote a GPOVM with the seed CM Σ by $\Pi^{\Sigma }=\left\{\Pi^{\Sigma }\left(\alpha \right)\right\}$ ([29,30]).

*A criterion for unsteerability of Gaussian states.* For arbitrary bipartite Gaussian states, the authors in [28] derived a linear matrix inequality that decides the question of steerability via GPOVMs.

**Theorem** **1.** *[28] Assume that $\rho_{AB}\in S\left(H_{A}⊗H_{B}\right)$ is any (m+n)-mode Gaussian state with CM Γ in Equation (1). Then $\rho_{AB}$ is unsteerable by the system A’s all GPOVMs if and only if*

(2) $$\begin{array}{c} \Gamma +0_{A}⊕iJ_{B}\geq 0, \end{array}$$

*where $J_{B}={⊕}_{n}\left(\begin{array}{cc} 0 & 1\\ -1 & 0 \end{array}\right)$.*

**Remark** **1.** *By Theorem 1, under the restriction of GPOVMs, Equation (2) is a necessary and sufficient condition for detecting the steering of Gaussian states. However, Equation (2) may not be sufficient for unsteerability of non-Gaussian bipartite states in continuous-variable systems. Recent works also revealed that there exist Gaussian states which are only steerable by suitable non-GPOVMs. In [25], the authors considered pseudospin measurements instead of GPOVMs for any two-mode Gaussian states and found that these observables are always less sensitive than conventional Gaussian observables for steering detection. Note that GPOVMs are accessible in laboratory by means of homodyne detections and Gaussian transformations. So in this paper, we restrict to GPOVMs when discussing the steering of Gaussian states.*

## 3. Steering Witness for Gaussian States and Their Comparability

In this section, we will first give a definition of steering witness for Gaussian states, and then discuss some properties of steering witness.

Denote by Sym(2N,R) the set of all real symmetric 2N×2N matrices. Note that a CM Γ can describe a physical quantum state if and only if it satisfies the bona fide uncertainty principle relation Γ+iJ≥0. Let CM(2(m+n),R) stand for the set of all CMs satisfying uncertainty principle relations in (m+n)-mode continuous-variable systems, that is,

(3) $$\begin{array}{c} \mathrm{CM}\left(2\left(m+n\right),R\right)=\{\Gamma \in \mathrm{Sym}\left(2\left(m+n\right),R\right):\Gamma \pm iJ\geq 0\;\;\mathrm{with}\;\;J={⊕}_{m+n}\left(\begin{array}{cc} 0 & 1\\ -1 & 0 \end{array}\right)\}. \end{array}$$

For the convenience, write N=m+n. Let

(4) $$\begin{array}{ccc} & & {\mathrm{US}}_{A|B}\left(2N,R\right)\\ & = & \{\Gamma \in \mathrm{CM}\left(2N,R\right):\Gamma +0_{A}⊕iJ_{B}\geq 0,\;0_{A}\in M_{2m}\left(R\right),\;J_{B}={⊕}_{n}\left(\begin{array}{cc} 0 & 1\\ -1 & 0 \end{array}\right)\}. \end{array}$$

We call the elements in ${\mathrm{US}}_{A|B}\left(2N,R\right)$ unsteerable CMs from A to B as Theorem 1. It is easily checked that ${\mathrm{US}}_{A|B}\left(2N,R\right)$ is a closed and convex set. The following result gives another property of ${\mathrm{US}}_{A|B}\left(2N,R\right)$.

**Proposition** **1.** *Assume that $\Gamma \in {\mathrm{US}}_{A|B}\left(2N,R\right)$. Then for any positive matrix P∈Sym(2N,R) and any scalar α>1, we have $\Gamma +P\in {\mathrm{US}}_{A|B}\left(2N,R\right)$ and $\alpha \Gamma \in {\mathrm{US}}_{A|B}\left(2N,R\right)$.*

**Proof.**  For any positive matrix P∈Sym(2N,R), by Equation (3), it is obvious that $\Gamma +P\in {\mathrm{US}}_{A|B}\left(2N,R\right)$. For any α>1, as

αΓ+iJ=(α−1)Γ+Γ+iJ>Γ+iJ≥0

and

$$\alpha \Gamma +0_{A}⊕iJ_{B}=\left(\alpha -1\right)\Gamma +\Gamma +0_{A}⊕iJ_{B}>\Gamma +0_{A}⊕iJ_{B}\geq 0,$$

we get $\alpha \Gamma \in {\mathrm{US}}_{A|B}\left(2N,R\right)$. □

Next, write

(5) $$\begin{array}{c} W_{A|B}\left(2N,R\right)=\left\{W\in \mathrm{Sym}\left(2N,R\right):\mathrm{Tr}\left(W\Gamma \right)\geq 1\;\;\mathrm{holds}\;\mathrm{for}\;\mathrm{all}\;\;\Gamma \in \;{\mathrm{US}}_{A|B}\left(2N,R\right)\right\}. \end{array}$$

We call any element *W* in $W_{A|B}\left(2N,R\right)$ the steering witness from A to B in (m+n)-mode bipartite continuous-variable systems with subsystems *A* and *B*, where N=m+n.

The following theorem gives a criterion of detecting steerability of any (m+n)-mode Gaussian states by steering witnesses.

**Theorem 2.**  *(Steering witness criterion) Assume that $\rho_{AB}\in S\left(H_{A}⊗H_{B}\right)$ is any (m+n)-mode Gaussian state with CM Γ defined by Equation (1). Then $\rho_{AB}$ is unsteerable from A to B if and only if Tr(WΓ)≥1 holds for all $W\in W_{A|B}\left(2\left(m+n\right),R\right)$.*

**Proof.**  For the “only if” part, if Tr(WΓ)≥1 holds for all $W\in W_{A|B}\left(2\left(m+n\right),R\right)$, by Equation (5), $\Gamma \in {\mathrm{US}}_{A|B}\left(2\left(m+n\right),R\right)$. It follows from Theorem 1 that $\rho_{AB}$ is unsteerable from A to B.

For the “if” part, on the contrary, suppose that $\rho_{AB}$ is steerable from A to B. Then Γ is steerable from A to B as Theorem 1, that is, $\Gamma \notin {\mathrm{US}}_{A|B}\left(2N,R\right)$ with N=m+n. Since the set ${\mathrm{US}}_{A|B}\left(2N,R\right)$ is convex and closed, by the Hahn-Banach theorem, there exists some $W_{1}\in \mathrm{Sym}\left(2N,R\right)$ such that

(6) $$\begin{array}{c} \mathrm{Tr}\left(W_{1}\Gamma^{\prime }\right)\geq m=\underset{\Gamma^{\prime }\in \;{\mathrm{US}}_{A|B}\left(2N,R\right)}{\inf}\mathrm{Tr}\left(W_{1}\Gamma^{\prime }\right)>\mathrm{Tr}\left(W_{1}\Gamma \right)\;\;\mathrm{for}\;\mathrm{all}\;\;\Gamma^{\prime }\in \;{\mathrm{US}}_{A|B}\left(2N,R\right). \end{array}$$

We claim m>0. Otherwise, assume m≤0. If $W_{1}$ is not positive, there is a negative eigenvalue $\lambda_{0}<0$ of $W_{1}$ with the corresponding eigenvector |ϕ〉. Take any η>0, any $\Gamma^{\prime }\in {\mathrm{US}}_{A|B}\left(2N,R\right)$ and let $\Gamma_{0}=\Gamma^{\prime }+\eta |\phi \rangle \langle \phi |$. By Proposition 1, $\Gamma_{0}\in {\mathrm{US}}_{A|B}\left(2N,R\right)$. Note that

$$\mathrm{Tr}\left(W_{1}\Gamma_{0}\right)=\mathrm{Tr}\left(W_{1}\Gamma^{\prime }\right)+\eta \mathrm{Tr}(W_{1}|\phi \rangle \langle \phi |)=\mathrm{Tr}\left(W_{1}\Gamma^{\prime }\right)+\lambda_{0}{\eta ∥|\phi \rangle ∥}^{2}\to -\infty \;\;\mathrm{whenever}\;\;\eta \to +\infty .$$

This means that, for sufficient large η>0, we have $\mathrm{Tr}\left(W_{1}\Gamma_{0}\right)\leq \mathrm{Tr}\left(W_{1}\Gamma \right)$, which yields a contradiction to Equation (6). Thus, $W_{1}$ is positive, and so $\mathrm{Tr}(W_{1}\Gamma^{\prime })\geq 0$. Further, we can conclude $\mathrm{Tr}(W_{1}\Gamma^{\prime })>0$. In fact, by Williamson normal form Theorem, for any CM $\Gamma^{\prime }\in \;{\mathrm{US}}_{A|B}\left(2N,R\right)$, there exists a symplectic matrix S∈Sym(2N,R) such that $S\Gamma^{\prime }S^{T}=\Gamma^{\prime \prime }={⊕}_{i=1}^{N}\left(\begin{array}{cc} v_{i} & 0\\ 0 & v_{i} \end{array}\right)$ with $v_{i}\geq 1$. So

$$\begin{array}{cc} \mathrm{Tr}(W_{1}\Gamma^{\prime })= & \mathrm{Tr}\left(W_{1}S^{-1}\Gamma^{\prime \prime }{\left(S^{T}\right)}^{-1}\right)=\mathrm{Tr}\left[{\left(S^{T}\right)}^{-1}W_{1}S^{-1}\Gamma^{\prime \prime }\right]\\ = & \mathrm{Tr}\left({\left(S^{T}\right)}^{-1}W_{1}S^{-1}\left(\Gamma^{\prime \prime }-I\right)\right)+\mathrm{Tr}\left({\left(S^{T}\right)}^{-1}W_{1}S^{-1}\right)>0. \end{array}$$

However, this leads to a contradiction with m≤0.

Hence m>0. By letting $W_{0}=\frac{W_{1}}{m}$ in Equation (6) yields

$$\mathrm{Tr}\left(W_{0}\Gamma^{\prime }\right)\geq 1>\mathrm{Tr}\left(W_{0}\Gamma \right)\;\;\mathrm{for}\;\mathrm{all}\;\;\Gamma^{\prime }\in \;{\mathrm{US}}_{A|B}\left(2N,R\right),$$

which implies $W_{0}\in W_{A|B}\left(2N,R\right)$ with $\mathrm{Tr}(W_{0}\Gamma )<1$, a contradiction. Therefore, Γ is unsteerable from A to B, and thus $\rho_{AB}$ is unsteerable from A to B. □

**Remark** **2.** *By Theorem 2, we see that, for any (m+n)-mode Gaussian state $\rho_{AB}\in S\left(H_{A}⊗H_{B}\right)$ with CM Γ, $\rho_{AB}$ is steerable from A to B if and only if there exists some $W_{0}\in W_{A|B}\left(2\left(m+n\right),R\right)$ such that $\mathrm{Tr}(W_{0}\Gamma )<1$.*

In the rest part, we will discuss the properties of steering witnesses. Given a steering witness $W\in W_{A|B}\left(2\left(m+n\right),R\right)$, denote the set of CMs detected by *W* by

$$D_{W}=\left\{\Gamma \in \mathrm{CM}\left(2\left(m+n\right),R\right):\mathrm{Tr}\left(W\Gamma \right)<1\right\}.$$

It is obvious that any two steering witnesses $W_{1}$ and $W_{2}$ have one of the following three relations:

(1) $D_{W_{1}}\subseteq D_{W_{2}}$ or $D_{W_{2}}\subseteq D_{W_{1}}$;

(2) $D_{W_{1}}⋂D_{W_{2}}=\emptyset$;

(3) $D_{W_{1}}⋂D_{W_{2}}\neq \emptyset$ and $D_{W_{i}}⊈D_{W_{j}}$, i≠j∈{1,2}.

**Definition** **1.** *For any two steering witnesses $W_{1}$ and $W_{2}$, we say that $W_{2}$ is finer than $W_{1}$, denote by $W_{1}≺W_{2}$, if $D_{W_{1}}\subseteq D_{W_{2}}$; and $W_{1}=W_{2}$ if $D_{W_{1}}=D_{W_{2}}$. Furthermore, we say that $W_{1}$ and $W_{2}$ are comparable if $W_{1}≺W_{2}$ or $W_{2}≺W_{1}$; otherwise, $W_{1}$ and $W_{2}$ are incomparable.*

*Particularly, for a steering witness W, we say that W is optimal if there is no other steering witness finer than W.*

The following result gives the relation of two comparable steering witnesses.

**Theorem** **3.** *Suppose that $W_{1},W_{2}\in W_{A|B}\left(2(m+n),R\right)$ are two steering witnesses with $W_{1}≺W_{2}$, and $\lambda =\underset{\Gamma_{1}\in D_{W_{1}}}{\inf}\frac{1-\mathrm{Tr}(W_{2}\Gamma_{1})}{1-\mathrm{Tr}(W_{1}\Gamma_{1})}.$ Then λ⩾1 and for any Γ∈CM(2(m+n),R), we have*

(i) *$\mathrm{Tr}(W_{2}\Gamma )⩽1$ if $\mathrm{Tr}(W_{1}\Gamma )=1$;*
(ii) *$\mathrm{Tr}\left(W_{2}\Gamma \right)⩽\mathrm{Tr}\left(W_{1}\Gamma \right)$ if $\mathrm{Tr}(W_{1}\Gamma )<1$;*
(iii) *$\mathrm{Tr}\left(W_{2}\Gamma \right)⩽\lambda \mathrm{Tr}\left(W_{1}\Gamma \right)$ if $\mathrm{Tr}(W_{1}\Gamma )>1$.*

**Proof.**  Assume that $W_{1},W_{2}\in W_{A|B}\left(2(m+n),R\right)$ are two steering witnesses with $W_{1}≺W_{2}$ and Γ∈CM(2(m+n),R).

(i) Assume that $\mathrm{Tr}(W_{1}\Gamma )=1$, but $\mathrm{Tr}(W_{2}\Gamma )>1$. Take any $\Gamma_{1}\in D_{W_{1}}$ and any positive number x>0. Write

$${\tilde{\Gamma }}_{x}=\frac{1}{1+x}\Gamma_{1}+\frac{x}{1+x}\Gamma .$$

Then ${\tilde{\Gamma }}_{x}\in \mathrm{CM}\left(2(m+n),R\right)$ and

$$\begin{array}{ccc} \mathrm{Tr}(W_{1}{\tilde{\Gamma }}_{x}) & = & \frac{1}{1+x}\mathrm{Tr}\left(W_{1}\Gamma_{1}\right)+\frac{x}{1+x}\mathrm{Tr}\left(W_{1}\Gamma \right)\\ & < & \frac{1}{1+x}+\frac{x}{1+x}=1. \end{array}$$

So ${\tilde{\Gamma }}_{x}\in D_{W_{1}}\subseteq D_{W_{2}}$ for all x>0.

On the other hand, note that $\mathrm{Tr}(W_{2}\Gamma_{1})<1$ as $\Gamma_{1}\in D_{W_{1}}\subseteq D_{W_{2}}$. Take any x>0 with $x>\frac{1-\mathrm{Tr}(W_{2}\Gamma_{1})}{\mathrm{Tr}(W_{2}\Gamma )-1}>0$. Then $x\mathrm{Tr}\left(W_{2}\Gamma \right)-x>1-\mathrm{Tr}\left(W_{2}\Gamma_{1}\right)$ and so

$$\mathrm{Tr}\left(W_{2}{\tilde{\Gamma }}_{x}\right)=\frac{1}{1+x}\mathrm{Tr}\left(W_{2}\Gamma_{1}\right)+\frac{x}{1+x}\mathrm{Tr}\left(W_{2}\Gamma \right)=\frac{\mathrm{Tr}\left(W_{2}\Gamma_{1}\right)+x\mathrm{Tr}\left(W_{2}\Gamma \right)}{1+x}>1.$$

This implies ${\tilde{\Gamma }}_{x}\notin D_{W_{2}}$ for such *x*, a contradiction.

(ii) Assume that $\mathrm{Tr}(W_{1}\Gamma )<1$. Letting $\tilde{\Gamma }=\frac{1}{\mathrm{Tr}(W_{1}\Gamma )}\Gamma$, then $\tilde{\Gamma }\in \mathrm{CM}\left(2(m+n),R\right)$ and

$$\mathrm{Tr}\left(W_{1}\tilde{\Gamma }\right)=\frac{\mathrm{Tr}(W_{1}\Gamma )}{\mathrm{Tr}(W_{1}\Gamma )}=1.$$

By (i), we have $\mathrm{Tr}(W_{2}\tilde{\Gamma })⩽1$, and so $\mathrm{Tr}\left(W_{2}\Gamma \right)⩽\mathrm{Tr}\left(W_{1}\Gamma \right)$.

(iii) If $\mathrm{Tr}(W_{1}\Gamma )>1$, by taking $a=\frac{\mathrm{Tr}(W_{1}\Gamma )-1}{\mathrm{Tr}\left(W_{1}\Gamma \right)-\mathrm{Tr}\left(W_{1}\Gamma_{1}\right)}$ and $b=\frac{1-\mathrm{Tr}(W_{1}\Gamma_{1})}{\mathrm{Tr}\left(W_{1}\Gamma \right)-\mathrm{Tr}\left(W_{1}\Gamma_{1}\right)}$ with $\Gamma_{1}\in D_{W_{1}}$, we have 0<a,b<1 and a+b=1. Write $\tilde{\Gamma }=a\Gamma_{1}+b\Gamma$. It is obvious that $\tilde{\Gamma }\in \mathrm{CM}\left(2(m+n),R\right)$ and

$$\mathrm{Tr}\left(W_{1}\tilde{\Gamma }\right)=a\mathrm{Tr}\left(W_{1}\Gamma_{1}\right)+b\mathrm{Tr}\left(W_{1}\Gamma \right)=1.$$

By (i), one gets $\mathrm{Tr}(W_{2}\tilde{\Gamma })⩽1$, that is, $a\mathrm{Tr}\left(W_{2}\Gamma_{1}\right)+b\mathrm{Tr}\left(W_{2}\Gamma \right)⩽1$. So

(7) $$\begin{array}{ccc} \mathrm{Tr}(W_{2}\Gamma ) & ⩽ & \frac{1-a\mathrm{Tr}(W_{2}\Gamma_{1})}{b}\\ & = & \frac{1-\frac{\mathrm{Tr}(W_{1}\Gamma )-1}{\mathrm{Tr}\left(W_{1}\Gamma \right)-\mathrm{Tr}\left(W_{1}\Gamma_{1}\right)}\cdot \mathrm{Tr}\left(W_{2}\Gamma_{1}\right)}{\frac{1-\mathrm{Tr}(W_{1}\Gamma_{1})}{\mathrm{Tr}\left(W_{1}\Gamma \right)-\mathrm{Tr}\left(W_{1}\Gamma_{1}\right)}}\\ & = & \frac{\mathrm{Tr}\left(W_{1}\Gamma \right)-\mathrm{Tr}\left(W_{1}\Gamma_{1}\right)-\mathrm{Tr}\left(W_{1}\Gamma \right)\cdot \mathrm{Tr}\left(W_{2}\Gamma_{1}\right)+\mathrm{Tr}\left(W_{2}\Gamma_{1}\right)}{1-\mathrm{Tr}(W_{1}\Gamma_{1})}\\ & = & \frac{\mathrm{Tr}\left(W_{1}\Gamma \right)\left[1-\mathrm{Tr}\left(W_{2}\Gamma_{1}\right)\right]-\left[\mathrm{Tr}\left(W_{1}\Gamma_{1}\right)-\mathrm{Tr}\left(W_{2}\Gamma_{1}\right)\right]}{1-\mathrm{Tr}(W_{1}\Gamma_{1})}\\ & ⩽ & \frac{\mathrm{Tr}\left(W_{1}\Gamma \right)\left[1-\mathrm{Tr}\left(W_{2}\Gamma_{1}\right)\right]}{1-\mathrm{Tr}(W_{1}\Gamma_{1})}. \end{array}$$

Note that the last inequality is due to (ii). Thus, Equation (7) implies

$$\frac{\mathrm{Tr}(W_{2}\Gamma )}{\mathrm{Tr}(W_{1}\Gamma )}⩽\frac{1-\mathrm{Tr}(W_{2}\Gamma_{1})}{1-\mathrm{Tr}(W_{1}\Gamma_{1})},$$

and hence

$$\frac{\mathrm{Tr}(W_{2}\Gamma )}{\mathrm{Tr}(W_{1}\Gamma )}⩽\underset{\Gamma_{1}\in D_{W_{1}}}{\inf}\frac{1-\mathrm{Tr}(W_{2}\Gamma_{1})}{1-\mathrm{Tr}(W_{1}\Gamma_{1})}=\lambda .$$

Finally, we will show λ≥1. In fact, for any $\Gamma_{1}\in D_{W_{1}}$, we have $\mathrm{Tr}(W_{1}\Gamma_{1})<1$, and by (ii), $\mathrm{Tr}\left(W_{2}\Gamma_{1}\right)⩽\mathrm{Tr}\left(W_{1}\Gamma_{1}\right)$. Thus, $1-\mathrm{Tr}\left(W_{2}\Gamma_{1}\right)⩾1-\mathrm{Tr}\left(W_{1}\Gamma_{1}\right)$, and so λ≥1. □

In the following theorem, we give a necessary and sufficient condition for two steering witnesses to be comparable.

**Theorem** **4.** *Suppose that $W_{1},W_{2}\in W_{A|B}\left(2\left(m+n\right),R\right)$ are two steering witnesses. Then $W_{1}≺W_{2}$ if and only if there exists some 0<a⩽1 and some positive matrix X∈Sym(2(m+n),R) satisfying Tr(XΓ)⩾1−a for all $\Gamma \in D_{W_{1}}$ such that*

$$W_{1}=aW_{2}+X.$$

**Proof.**  Assume that $W_{1},W_{2}\in W_{A|B}\left(2\left(m+n\right),R\right)$ are two steering witnesses. If there exists some 0<a⩽1 and some positive matrix X∈Sym(2(m+n),R) with Tr(XΓ)⩾1−a for all $\Gamma \in D_{W_{1}}$ such that $W_{1}=aW_{2}+X,$ then, for any $\Gamma \in D_{W_{1}}$, we have

$$1>\mathrm{Tr}\left(W_{1}\Gamma \right)=a\mathrm{Tr}\left(W_{2}\Gamma \right)+\mathrm{Tr}\left(X\Gamma \right)\geq a\mathrm{Tr}\left(W_{2}\Gamma \right)+1-a.$$

It follows that $\mathrm{Tr}(W_{2}\Gamma )<1$. So $\Gamma \in D_{W_{2}}$. By Definition 1, one obtains $W_{1}≺W_{2}$.

Conversely, if $W_{1}≺W_{2}$, by taking $\lambda =\underset{\Gamma_{1}\in D_{W_{1}}}{\inf}\frac{1-\mathrm{Tr}(W_{2}\Gamma_{1})}{1-\mathrm{Tr}(W_{1}\Gamma_{1})}$ and by Theorem 3, we have $\frac{1-\mathrm{Tr}(W_{2}\Gamma_{1})}{1-\mathrm{Tr}(W_{1}\Gamma_{1})}⩾\lambda \geq 1$ for all $\Gamma_{1}\in D_{W_{1}}$, that is,

(8) $$\begin{array}{c} \mathrm{Tr}\left[\left(\lambda W_{1}-W_{2}\right)\Gamma_{1}\right]⩾\lambda -1\;\;\mathrm{for}\;\mathrm{all}\;\;\Gamma_{1}\in D_{W_{1}}. \end{array}$$

On the other hand, for any $\Gamma \in \mathrm{CM}\left(2\left(m+n\right),R\right)\setminus D_{W_{1}}$, by Theorem 3 (iii), one has $\mathrm{Tr}\left(W_{2}\Gamma \right)⩽\lambda \mathrm{Tr}\left(W_{1}\Gamma \right)$, that is,

(9) $$\begin{array}{c} \mathrm{Tr}\left[\left(\lambda W_{1}-W_{2}\right)\Gamma \right]⩾0\;\;\mathrm{for}\;\mathrm{all}\;\;\Gamma \in \mathrm{CM}\left(2\left(m+n\right),R\right)\setminus D_{W_{1}}. \end{array}$$

Combining Equations (8) and (9) gives

(10) $$\begin{array}{c} \mathrm{Tr}\left[\left(\lambda W_{1}-W_{2}\right)\Gamma \right]⩾0\;\;\mathrm{for}\;\mathrm{all}\;\;\Gamma \in \mathrm{CM}\left(2\left(m+n\right),R\right). \end{array}$$

Now, let $X=W_{1}-aW_{2}$ with $a=\frac{1}{\lambda }$. Obviously, 0<a⩽1 and Tr(XΓ)⩾1−a holds for all $\Gamma \in D_{W_{1}}$ by Equation (8).

Finally, if *X* is not positive, then there is a negative eigenvalue $\mu_{0}<0$ of *X* with the corresponding eigenvector |ζ〉. Take any η>0, any Γ∈CM(2(m+n),R) and let $\Gamma_{0}=\Gamma +\eta |\zeta \rangle \langle \zeta |$. Obviously, $\Gamma_{0}\in \mathrm{CM}\left(2\left(m+n\right),R\right)$. Note that

$$\mathrm{Tr}\left(X\Gamma_{0}\right)=\mathrm{Tr}\left(X\Gamma \right)+\eta \mathrm{Tr}(X|\zeta \rangle \langle \zeta |)=\mathrm{Tr}\left(X\Gamma \right)+\mu_{0}{\eta ∥|\zeta \rangle ∥}^{2}\to -\infty \;\;\mathrm{whenever}\;\;\eta \to +\infty .$$

Also note that

Tr(XΓ)≥0forallΓ∈CM(2(m+n),R)

by Equation (10). These yield a contradiction. So *X* is positive.

The proof of the theorem is finished. □

For the optimality of steering witnesses, we have

**Theorem** **5.** *Suppose that $W\in W_{A|B}\left(2\left(m+n\right),R\right)$ is a steering witness. Then W is optimal if and only if for any λ⩾1 and any positive matrix X∈Sym(2(m+n),R) satisfying Tr(XΓ)⩾λ−1 for all $\Gamma \in D_{W}$, $W^{\prime }=\lambda W-X$ is not a steering witness.*

**Proof.**  The “if” part is obvious by Theorem 4.

For the “only if” part, assume that there is some $\lambda_{0}⩾1$ and some positive matrix $X_{0}\in \mathrm{Sym}\left(2\left(m+n\right),R\right)$ satisfying $\mathrm{Tr}\left(X_{0}\Gamma \right)⩾\lambda_{0}-1$ for all $\Gamma \in D_{W}$ such that $W^{\prime }=\lambda_{0}W-X_{0}$ is a steering witness. Then $W=\frac{1}{\lambda_{0}}W^{\prime }+\frac{1}{\lambda_{0}}X_{0}$, where $\frac{1}{\lambda_{0}}\leq 1$ and $\frac{1}{\lambda_{0}}X_{0}\geq 0$ with $\mathrm{Tr}\left(\frac{1}{\lambda_{0}}X_{0}\Gamma \right)⩾1-\frac{1}{\lambda_{0}}$ for all $\Gamma \in D_{W}$. By Theorem 4 again, $W≺W^{\prime }$. A contradiction. □

Finally, we discuss the question when different steering witnesses can detect some common steering CMs.

**Theorem** **6.** *For any two steering witnesses $W_{1},W_{2}\in W_{A|B}\left(2\left(m+n\right),R\right)$, we have $D_{W_{1}}\cap D_{W_{2}}=\emptyset$ if and only if there exists some 0<λ<1 such that $\lambda W_{1}+\left(1-\lambda \right)W_{2}\notin W_{A|B}\left(2\left(m+n\right),R\right)$.*

To prove the theorem, two lemmas are needed.

**Lemma** **1.** *Suppose that $W_{1},W_{2}\in W_{A|B}\left(2\left(m+n\right),R\right)$ are steering witnesses with $W_{1}≺W_{2}$. If $W\left(a,b\right)=aW_{1}+bW_{2}$ with a,b>0 and a+b=1, then $W_{1}≺W\left(a,b\right)≺W_{2}$.*

**Proof.**  For two steering witnesses $W_{1},W_{2}\in W_{A|B}\left(2\left(m+n\right),R\right)$ with $W_{1}≺W_{2}$, we have $\mathrm{Tr}(W_{1}\Gamma )<1$ for all $\Gamma \in D_{W_{1}}$. By Theorem 3(ii), $\mathrm{Tr}\left(W_{2}\Gamma \right)⩽\mathrm{Tr}\left(W_{1}\Gamma \right)$, and so $\mathrm{Tr}(\left(aW_{1}+bW_{2}\right)\Gamma )<a+b=1$. This means $\Gamma \in D_{W(a,b)}$, that is, $W_{1}≺W\left(a,b\right)$.

In addition, if $\Gamma \notin D_{W_{2}}$, then $\mathrm{Tr}(W_{2}\Gamma )\geq 1$ and $\mathrm{Tr}(W_{1}\Gamma )\geq 1$ as $W_{1}≺W_{2}$. Thus

$$\mathrm{Tr}\left(W\left(a,b\right)\Gamma \right)=a\mathrm{Tr}\left(W_{1}\Gamma \right)+b\mathrm{Tr}\left(W_{2}\Gamma \right)\geq 1.$$

This implies $\Gamma \notin D_{W(a,b)}$. So $W\left(a,b\right)≺W_{2}$. □

**Lemma** **2.** *Assume that $W,W_{1},W_{2}\in W_{A|B}\left(2\left(m+n\right),R\right)$ are steering witnesses. If $D_{W_{1}}\cap D_{W_{2}}=\emptyset$ and $D_{W}\subseteq D_{W_{1}}\cup D_{W_{2}}$, then either $D_{W}\subseteq D_{W_{1}}$ or $D_{W}\subseteq D_{W_{2}}$.*

**Proof.**  Assume, on the contrary, that $D_{W}\cap D_{W_{1}}\neq \emptyset$ and $D_{W}\cap D_{W_{2}}\neq \emptyset$. Take $\Gamma_{i}\in D_{W}\cap D_{W_{i}}$ for i=1,2. Write

$$\left[\Gamma_{1},\Gamma_{2}\right]=\left\{\Gamma_{t}=t\Gamma_{1}+\left(1-t\right)\Gamma_{2},0\leq t\leq 1\right\}.$$

Note that $D_{W}$ is a convex set. So $\left[\Gamma_{1},\Gamma_{2}\right]\subseteq D_{W}\subseteq D_{W_{1}}\cup D_{W_{2}}$ and thus $\left[\Gamma_{1},\Gamma_{2}\right]\subseteq \left(D_{W}\cap D_{W_{1}}\right)\cup \left(D_{W}\cap D_{W_{2}}\right)$. Hence there exists some $0<t_{0}<1$ such that $\left\{\Gamma_{t}:0\leq t<t_{0}\right\}\subseteq D_{W_{2}}$ and $\left\{\Gamma_{t}:t_{0}<t\leq 1\right\}\subseteq D_{W_{1}}$. If $\Gamma_{t_{0}}\in D_{W_{2}}$, then $\mathrm{Tr}(W_{2}\Gamma_{t_{0}})<1$, and for sufficiently small ε>0, we have

$$1\leq \mathrm{Tr}\left(W_{2}\Gamma_{t_{0}+\varepsilon }\right)=\mathrm{Tr}\left(W_{2}\Gamma_{t_{0}}\right)+\varepsilon (\mathrm{Tr}\left(\left(W_{2}\Gamma_{1}\right)-\mathrm{Tr}\left(W_{2}\Gamma_{2}\right)\right)<1.$$

A contradiction. Similarly, if $\Gamma_{t_{0}}\in D_{W_{1}}$, by considering $\Gamma_{t_{0}-\varepsilon }$ for sufficiently small ε>0, one can also obtain a contradiction.

Therefore, $D_{W}\subseteq D_{W_{1}}$ or $D_{W}\subseteq D_{W_{2}}$. The proof is completed. □

**Proof of Theorem** **6.**  Take any two steering witnesses $W_{1},W_{2}\in W_{A|B}\left(2\left(m+n\right),R\right)$. If there exists some 0<λ<1 such that $W=\lambda W_{1}+\left(1-\lambda \right)W_{2}$ is not a steering witness, then $D_{W_{1}}\cap D_{W_{2}}\subseteq D_{W}=\emptyset$, that is, $D_{W_{1}}\cap D_{W_{2}}=\emptyset$.

For the “only if” part, assume that $D_{W_{1}}\cap D_{W_{2}}=\emptyset$ and $W_{\lambda }=\lambda W_{1}+\left(1-\lambda \right)W_{2}\in W_{A|B}\left(2\left(m+n\right),R\right)$ for all 0<λ<1. Then $D_{W_{\lambda }}\subseteq D_{W_{1}}\cup D_{W_{2}}$. Since $D_{W_{1}}\cap D_{W_{2}}=\emptyset$, by Lemma 2, we have either $D_{W_{\lambda }}\subseteq D_{W_{1}}$ or $D_{W_{\lambda }}\subseteq D_{W_{2}}$. When λ varies from 0 to 1 continuously, $D_{W_{\lambda }}$ varies from $D_{W_{2}}$ to $D_{W_{1}}$ continuously. Denote $\lambda_{0}=\sup\left\{\lambda \in \left(0,1\right):D_{W_{\lambda }}\subseteq D_{W_{2}}\right\}$. If $D_{W_{\lambda_{0}}}\subseteq D_{W_{2}}$, then there must be exist some ε with $0<\varepsilon <1-\lambda_{0}$ such that $W_{\lambda_{0}+\varepsilon }$ is not a steering witness, that is, $D_{W_{\lambda_{0}+\varepsilon }}=\emptyset$. Otherwise, for all ε with $0<\varepsilon <1-\lambda_{0}$, we have $D_{W_{\lambda_{0}+\varepsilon }}\neq \emptyset$. Since $D_{W_{\lambda_{0}}}\subseteq D_{W_{2}}$ and $D_{W_{\lambda_{0}+\varepsilon }}\subseteq D_{W_{1}}$, for all $\gamma \in D_{W_{\lambda_{0}}}$, one has $\mathrm{Tr}(W_{\lambda_{0}}\gamma )<1$ and

$$1\leq \mathrm{Tr}\left(W_{\lambda_{0}+\varepsilon }\gamma \right)=\mathrm{Tr}\left(W_{\lambda_{0}}\gamma \right)+\varepsilon \left(\mathrm{Tr}\left(W_{1}\gamma \right)-\mathrm{Tr}\left(W_{2}\gamma \right)\right)<1$$

for sufficiently small ε>0, a contradiction. Hence $D_{W_{\lambda_{0}}}⊈D_{W_{2}}$.

Similarly, one can show $D_{W_{\lambda_{0}}}⊈D_{W_{1}}$. So there exists some 0<λ<1, such that $\lambda W_{1}+\left(1-\lambda \right)W_{2}$ is not a steering witness. The proof is finished. □

## 4. Conclusions

Quantum EPR steering is an important quantum resource. It is a fundamental and important question of how to detect steerability of quantum states. In this paper, we investigated steering witnesses of Gaussian states in continuous-variable systems. We give a definition of steering witnesses by covariance matrices of quantum states, and then present a steering witness criterion of any (m+n)-mode Gaussian state to be unsteerable by the Hahn-Banach theorem. In addition, the conditions for any two steering witnesses to be comparable and the optimality of steering witnesses are also discussed. Our investigations may highlight further researches on steering witnesses.

## Data Availability

Data sharing not applicable.
